# Transcriptome Analysis Reveals MFGE8-HAPLN3 Fusion as a Novel Biomarker in Triple-Negative Breast Cancer

**DOI:** 10.3389/fonc.2021.682021

**Published:** 2021-06-15

**Authors:** Meng-Yuan Wang, Man Huang, Chao-Yi Wang, Xiao-Ying Tang, Jian-Gen Wang, Yong-De Yang, Xin Xiong, Chao-Wei Gao

**Affiliations:** Department of Breast Surgery, Chongqing University Three Gorges Hospital, Chongqing, China

**Keywords:** triple-negative breast cancer, *MFGE8-HAPLN3*, biomarker, target, precision treatment, fusion

## Abstract

**Background:**

Triple-negative breast cancer (TNBC) is a highly aggressive cancer with poor prognosis. The lack of effective targeted therapies for TNBC remains a profound clinical challenge. Fusion transcripts play critical roles in carcinogenesis and serve as valuable diagnostic and therapeutic targets in cancer. The present study aimed to identify novel fusion transcripts in TNBC.

**Methods:**

We analyzed the RNA sequencing data of 360 TNBC samples to identify and filter fusion candidates through SOAPfuse and ChimeraScan analysis. The characteristics, including recurrence, fusion type, chromosomal localization, TNBC subgroup distribution, and clinicopathological correlations, were analyzed in all candidates. Furthermore, we selected the promising fusion transcript and predicted its fusion type and protein coding capacity.

**Results:**

Using the RNA sequencing data, we identified 189 fusion transcripts in TNBC, among which 22 were recurrent fusions. Compared to para-tumor tissues, TNBC tumor tissues accumulated more fusion events, especially in high-grade tumors. Interestingly, these events were enriched at specific chromosomal loci, and the distribution pattern varied in different TNBC subtypes. The vast majority of fusion partners were discovered on chromosomes 1p, 11q, 19p, and 19q. Besides, fusion events mainly clustered on chromosome 11 in the immunomodulatory subtype and chromosome 19 in the luminal androgen receptor subtype of TNBC. Considering the tumor specificity and frameshift mutation, we selected *MFGE8-HAPLN3* as a novel biomarker and further validated it in TNBC samples using PCR and Sanger sequencing. Further, we successfully identified three types of *MFGE8-HAPLN3* (E6-E2, E5-E3, and E6-E3) and predicted the ORF of E6-E2, which could encode a protein of 712 amino acids, suggesting its critical role in TNBC.

**Conclusions:**

Improved bioinformatic stratification and comprehensive analysis identified the fusion transcript *MFGE8-HAPLN3* as a novel biomarker with promising clinical application in the future.

## Introduction

Triple-negative breast cancer (TNBC) lacks the expression of estrogen receptor (ER), progesterone receptor (PR), and human epidermal growth factor receptor 2 (HER2) and accounts for 10% to 20% of the newly diagnosed breast cancer cases (1, 2). While recognized as the most aggressive breast cancer subtype, women with TNBC have larger tumors, a higher rate of node positivity, and an increased likelihood of distant recurrence (3). Chemotherapy is yet the primary mode of treatment for early and advanced disease owing to the lack of molecular targets for therapy. Although several targeted therapies are showing potent efficacy, patients with TNBC have a worse prognosis compared to those with other breast cancer subtypes (4–6).

Fusion genes, formed by chromosomal rearrangements that juxtapose two different genes, can lead to abnormal activation of one or both genes and drive tumorigenesis (7). Based on the development of sequencing technologies and bioinformatics approaches, a number of fusion genes have been revealed over the past few decades (8, 9). Recently, fusion genes involving *ZNF384* have been identified in B-cell precursor acute lymphoblastic leukemia; eight fusion partners have been reported for the *ZNF384* gene. Moreover, the clinical features of patients depend on the functional defect of the fusion partner gene of *ZNF384* (10). Fusion transcripts, chimeric RNAs encoded by fusion genes or generated through subsequent cis-splicing and trans-splicing of mRNA in the absence of DNA rearrangements, serve as frequent drivers in a wide range of tumor types (11–13). Many fusion transcripts preferentially present in tumors compared to normal tissues, and contribute to tumor progression by enhancing cell proliferation and invasion (14). Significantly, the discovery that cancers harbor specific fusion genes or transcripts has enhanced the development of novel diagnostic and therapeutic strategies. For instance, tyrosine kinase inhibitors, such as imatinib, have been highly effective in the treatment of cancers harboring kinase fusions in leukemia and other cancers (15, 16).

Previous studies demonstrated that fusion candidates are involved in the tumorigenesis and progression of breast cancer. However, recurrent gene fusions have only been identified in rare subtypes of breast cancer. For example, some secretory carcinomas of the breast are driven by an *ETV6-NTRK3* fusion resulted from t(12;15)(p13;q25) chromosomal translocation (17). Similarly, adenoid cystic carcinomas of the breast are largely driven by a t(6;9)(q22-23;p23-24) translocation that forms a *MYB-NFIB* gene fusion (18). In addition to fusion genes, several fusion transcripts specifically present in breast cancer have been identified, including *CRTC1-MAML2*, *SCNN1A-TNFRSF1A*, and *CTSD-IFITM10* (19–21). Interestingly, some of the recurrent fusion transcripts encode membrane proteins, raising the possibility that they are breast cancer-specific cell surface markers and could be targeted by antibody drug conjugates (19). However, only little is known about fusion genes or transcripts in TNBC.

In the present study, we comprehensively revealed the landscape of fusion transcripts in TNBC. We also investigated the characteristics, including recurrence, fusion type, clinical relevance, and subgroup distribution. We discovered a novel fusion transcript *MFGE8-HAPLN3* in TNBC, highlighting the potential implications of fusion transcripts in cancer development and response to therapy.

## Materials and Methods

### Patient Cohorts

RNA-seq data used in the current study were downloaded from the Gene Expression Omnibus (GEO) (GSE118527) (https://www.ncbi.nlm.nih.gov/geo/) and The National Omics Data Encyclopedia (NODE) (OEP000155) (http://www.biosino.org/node). The RNA-seq data of 360 tumor tissues and 88 adjacent normal breast tissues were obtained. In addition, the corresponding clinicopathological characteristics, including age, histological type of the tumor, tumor size, lymph node status, histological grade, clinical stage and ER, PR, HER2, and Ki67 status, were collected (5). Our study was approved by the independent Ethics Committee/Institutional Review Board of Chongqing University Three Gorges Hospital.

### Identification of Fusion Transcripts in TNBC

RNA sequencing data were analyzed using ChimeraScan (22) and SOAPfuse (23) algorithms, which identify gene fusion candidates by detecting read pairs discordantly mapped to two different genes. The RNA-seq data of 448 samples (360 tumor samples and 88 adjacent normal breast tissues) were analyzed in random order. Fusion candidate that could be detected in at least one sample by two different algorithms was defined as double-positive fusion transcript (DPFT). We compared the frequency of fusion between tumor tissues and normal breast tissues and also described the characteristics, such as recurrence, fusion type, protein-coding capacity, chromosomal localization, and TNBC subgroup distribution, in all candidates. Subsequently, patients with DPFT were divided into low-fusion transcripts (low FTs) and high-fusion transcripts (high FTs) groups to evaluate the correlation between the expression level of fusion candidates and the clinicopathological features. Patients with fusion transcripts <4 were grouped as low FTs, while those with fusion transcripts ≥ 4 were grouped as high FTs.

### Promising Novel Fusion Screening

We selected promising fusion transcripts according to the following conditions: (1) recurrent fusion transcripts; (2) fusion transcripts with different functions formed by promoter swapping in the non-coding regions or frameshift in the coding regions; (3) fusion transcripts whose partner genes are associated with tumor; (4) fusion transcripts overexpressed in tumor tissue but unexpressed or expressed in low amounts in the adjacent normal breast tissue. We further predicted the open reading frame (ORF) and protein-coding capacity of the fusion transcripts according to the nucleotide sequence.

### Statistical Analyses

The data distribution was characterized by frequency tabulation and summary statistics. The data were examined for normality using the Shapiro-Wilk test. The continuous variables with normal distribution were assessed using the *t*-test or one-way analyses of variance (ANOVA), while the variables that did not meet the normal distribution were analyzed using the Mann-Whitney Wilcoxon test or Kruskal-Wallis test; Pearson’s Chi-square test or Fisher’s exact test was used to compare the categorical variables. In addition, the correlations were analyzed using the Pearson or Spearman test according to the normality of the distribution. All tests were two-sided, and P<0.05 indicated statistical significance. All statistical analyses were performed using SPSS 25.0 or R Studio (version 1.1.463, www.R-project.org).

## Results

### Fusion Transcripts Screening

**Figure 1:**
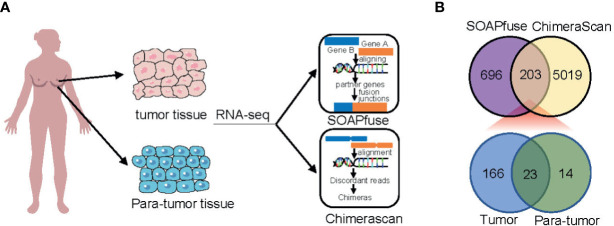
Identification of fusion transcripts in TNBC. **(A)** Flowchart of fusion transcripts screening. **(B)** Venn diagram summarizing the fusion transcripts detected using ChimeraScan and SOAPfuse algorithms.

To detect fusion transcripts, RNA-seq data from a set of 448 frozen samples (360 tumor tissues and 88 adjacent normal tissues) were analyzed (**Figure 1A**). A total of 203 fusion candidates, confirmed to be DPFT, were identified from 123 samples (66 tumor samples and 57 adjacent normal tissues) using both ChimeraScan and SOAPfuse algorithms (**Figure 1B**). Among these, 166 fusion transcripts were tumor-specific, while only 23 fusion transcripts were present in both tumor tissues and adjacent normal tissues (**Figure 1B** and **Supplementary Table 1**). Next, we investigated the frequency of fusion events in each sample. Compared to the adjacent normal tissues, more candidates were detected in TNBC tissues (mean fusions per sample: 4.106 *vs.* 1.807, P<0.05, **Supplementary Table 2**). These findings suggested that TNBC tissues are more likely to harbor fusion events compared to adjacent normal tissues.

### Characteristics of Selected Fusion Transcripts

**Figure 2:**
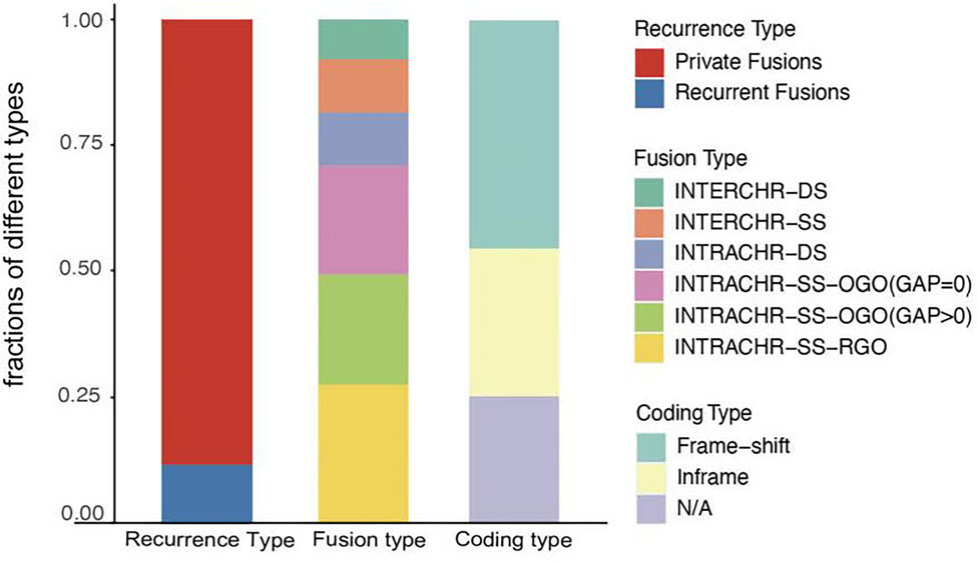
Characterization of fusion transcripts detected in TNBC. N/A, noncoding.

Next, we analyzed the characteristics, including recurrence, fusion type, and protein-coding capacity of 189 candidates (**Figure 2**) and found that 11.6% (22/189) of all candidates were present in two or more tumor tissues. In order to elucidate the mechanism, the fusion type was further analyzed using SOAPfuse algorithm. According to the relative locations of fusion partner genes, five types of fusion transcripts (INTERCHR-DS, INTERCHR-SS, INTRACHR-DS, INTRACHR-SS-OGO, INTRACHR-SS-RGO) were identified. These transcripts involving sequences from the same chromosomes constituted 81.5% of the total, thereby indicating that the majority of the fusion events occur at the transcriptional level. We also inferred the protein-coding capacity based on the junction sequence. Among these, 141 candidates (including 86 frameshift and 55 in-frame variants) could encode chimeric proteins, suggesting that the majority of the fusion transcripts have the potential to encode functional proteins.

### Clinical Association of Fusion Transcript Frequency in TNBC

**Figure 3:**
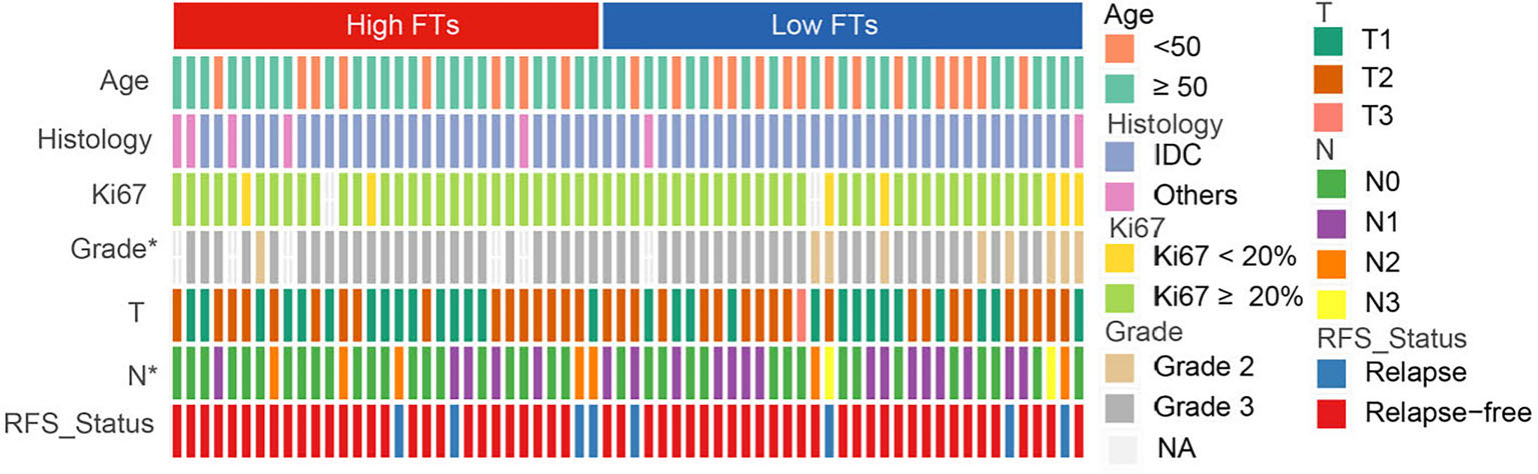
Association between frequency of fusion events and clinicopathological features. FTs, fusion transcripts; high FTs, patients whose fusion transcripts equal to or greater than 4; low FTs, patients whose fusion transcripts less than 4; IDC, infiltrated ductal carcinoma; NA, not available. *p < 0.05.

To explore the association between the frequency of fusion events and clinicopathological features, we divided 66 patients with TNBC into high and low FTs groups according to the number of fusion transcripts. The difference in the clinicopathological factors, including age, histological type of the tumor, Ki67 status, and clinical stage, was not statistically significant between two groups (**Figure 3**). Notably, an apparent discrepancy in the frequency of fusion events in different pathological grades (P<0.05) indicated that fusion events preferentially expressed in high-grade tumors.

### Subtype-Specific Chromosome Distribution of Fusion Transcripts

**Figure 4:**
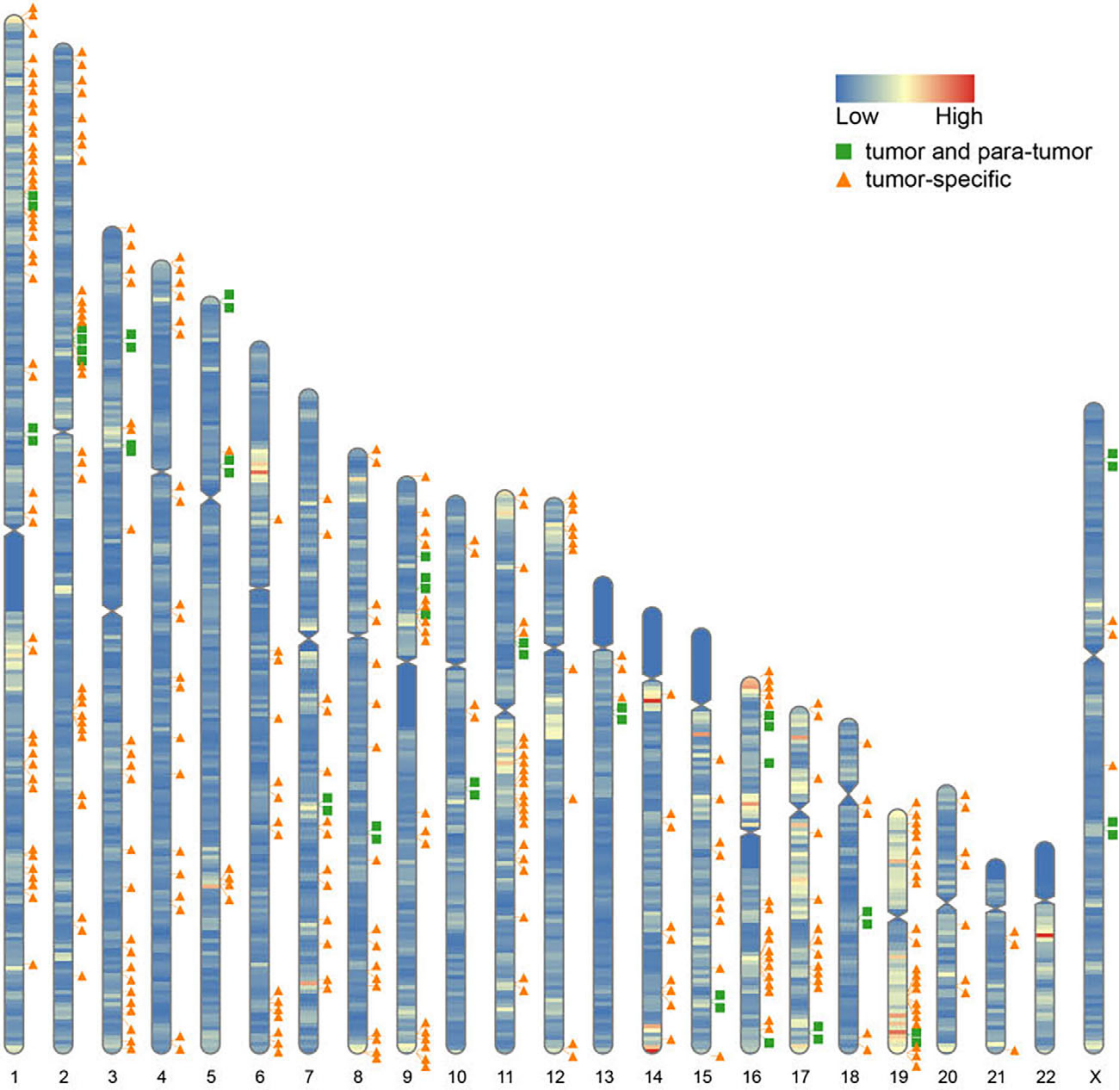
Chromosomal distribution of fusion partner genes. Green box indicates fusion partner genes in both tumor and para-tumor tissues; Orange triangle indicates fusion partner genes especially in tumor tissues.

Furthermore, we found that fusion transcripts were not randomly distributed on chromosomes (**Figure 4**). A disproportionately large number of fusion partner genes were detected in some specific chromosomes (hot spot region, chromosome arms 1p, 2p, 3q, 9p, 11q, 17q, 19p and 19q). Conversely, only a few fusion partner genes appeared in the cold spot region (chromosomes 10, 13, 21, and 22).

**Figure 5:**
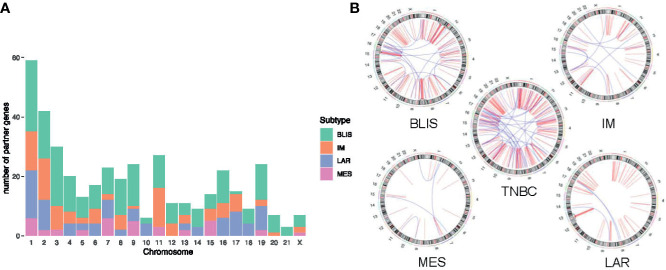
Distribution of fusion partner genes in different molecular subtypes of TNBC. **(A)** Frequency of fusion partner genes in each chromosome. **(B)** Relative locations of fusion partner genes. Intrachromosomal fusions are shown in red, and interchromosomal fusions are shown in blue. TNBC, triple-negative breast cancer; BLIS, basal-like immune-suppressed; IM, immunomodulatory; LAR, luminal androgen receptor; MES, mesenchymal-like.

Jiang and colleagues preciously presented a multiomics profiling of 465 Chinese patients with TNBCs, thus providing a large data set of comprehensively profiled TNBCs (5). Herein, they classified the TNBCs into four mRNA subtypes with distinct molecular features: 1) luminal androgen receptor (LAR), 2) immunomodulatory (IM), 3) basal-like immune-suppressed (BLIS), and 4) mesenchymal-like (MES). Then, the chromosome distribution of fusion transcripts across different molecular subtypes was characterized (**Figure 5**). The fusion accumulation at chromosome 11 in IM subtype and chromosomes 17 and 19 in LAR subtype suggested a subtype specificity of fusion candidates. Interestingly, our analysis indicated that fusion events were enriched in chromosomes 7, 9, and 15 in MES subtype and were rare in chromosomes 2 and 3. In addition, the frequency of fusion transcripts was significantly different between subtypes (**Supplementary Table 3**). BLIS subtype contained a vast majority of candidates detected in TNBC samples. Conversely, fusion transcripts were rare in MES subtype. Overall, our analysis demonstrated that fusion transcripts in specific chromosomes might exert isoform-specific roles in different molecular subtypes of TNBC.

### *MFGE8-HAPLN3* Fusion in TNBC

**Figure 6:**
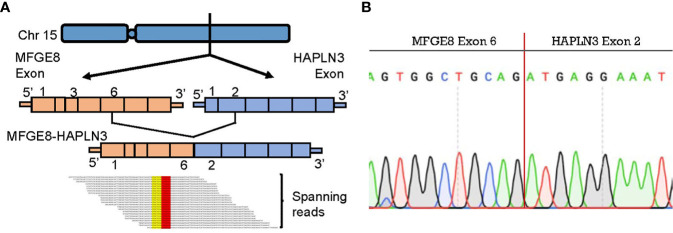
*MFGE8-HAPLN3* fusion in TNBC. **(A)** Schematic representation of the *MFGE8-HAPLN3* fusion transcript identified in TNBC. **(B)** PCR and Sanger sequencing verified the *MFGE8-HAPLN3* fusion.

To further explore biomarkers of clinical relevance, we selected fusion transcripts from 189 observed fusion candidates, according to the criteria described above. Finally, *MFGE8-HAPLN3* (16.7% in TNBC tumor tissues *vs.* 3.5% in adjacent normal breast tissues) was screened out. Milk fat globule-EGF factor 8 protein (*MFGE8*) and Hyaluronan And Proteoglycan Link Protein 3 (*HAPLN3*) were both located on the long arm of chromosome 15 (15q26.1, **Figure 6A**), suggesting that this fusion could be attributed to transcriptional read-through.

According to PCR and Sanger sequencing, three types of *MFGE8-HAPLN3* fusions, including E6-E2 (most frequently, **Figure 6B**), E5-E3, and E6-E3, were identified. Further, we successfully predicted an ORF of *MFGE8-HAPLN3* (E6-E2) that could encode a protein of 712 amino acids. Collectively, these findings suggested that *MFGE8-HAPLN3* fusion exists in TNBC samples and plays a critical role in TNBC. The predicted ORF of *MFGE8-HAPLN3* and its function *in vivo* have yet to be unambiguously characterized and need to be verified in further exploration.

## Discussion

Gene fusions/transcripts are important driver events in neoplasia and serve as valuable diagnostic biomarkers and therapeutic targets in cancer. Aberrant fusions have been widely described in multiple tumor types, such as non-small-cell lung cancer (24) and lymphoid neoplasms (25). The current study comprehensively explored the fusion transcripts in TNBC. We discovered 189 novel fusion transcripts and identified *MFGE8-HAPLN3* as a potential biomarker in TNBC.

As reported previously, tumor samples displayed more fusions than normal tissues. In accordance with these studies, our analysis revealed that TNBC tumor tissues accumulated a significantly higher number of fusion events than adjacent normal breast tissues. Typically, tumor characterized by a high frequency of fusion has been implicated in chromosomal instability (26, 27). Structural chromosome rearrangements effectuate the exchange of DNA sequences, inducing cancer cell progression (28). For example, Mitani et al. contended that *MYB-NFIB* gene fusion promoted the aggressive behavior in adenoid cystic carcinoma (29). In addition to chromosomal instability, aberrant regulation of the transcriptional process may also result in fusion transcripts. Herein, we observed a lot of fusion events involving genes on the same chromosome, suggesting that abundant fusions occur at the transcriptional level. An increasing number of studies supported that the disturbance of transcriptional regulation in transcription initiation, alternative splicing, or post-transcriptional modifications also can generate transcriptional read-through and contribute to tumor development (30–32). It is noteworthy that RNA processing of transcripts encoded by fusion genes makes splicing process significantly more complex. Regulatory sites nearby and surrounding the fusion junction sites are essential to the compatibility between sequences and spliceosome, which is necessary to canonical and alternative splicing (33). However, we could not differentiate between the transcriptome-level and genome-level changes. Fusion proteins that encoded by either fusion genes or fusion transcripts play nearly identical biological roles. It will be interesting to further explore the mechanism generating these chimeras.

To better understand the mechanism of novel fusion transcripts, we explored the fusion partner genes’ distribution and found they were non-randomly distributed on the chromosome. A majority of partners were enriched on chromosomes 1p, 2p, 9p, 11q, 19p, and 19q. Chromosome 19 has been reported as a fusion “hotspot” for TNBC. All fusion partners in TNBC mapped to clusters were located in the vicinity of 19p13 or 19q13 (34). In addition, we observed a discrepancy in chromosomal distribution between different TNBC subtypes. Fusion events clustered on chromosome 11 in the IM subtype. Differently, fusions are mainly located on chromosomes 17 and 19 in the LAR subtype. These findings demonstrated a strong functional association between the formation of fusion events and the TNBC subtypes.

Furthermore, we demonstrated the presence of a recurrent fusion transcript *MFGE8-HAPLN3*. *MFGE8*, and *HAPLN3* were neighboring genes on the same strand, suggesting that the fusion may be largely attributed to transcriptional read-through. Next, we successfully predicted its ORF and corresponding chimeric proteins. MFGE8 is a kind of soluble glycoprotein found in vertebrates and was initially discovered as a critical component of the milk fat globule. MFGE8 has been studied as a key regulator of various biological functions, including phagocytic removal of apoptotic cells in many tissues, the maintenance of intestinal epithelial homeostasis, and the promotion of mucosal healing (35). Recent studies have clarified the effect of MFGE8 on cell survival, adhesion, and migration in a wide spectrum of tumor types, such as ovarian cancer (36) and hepatocellular carcinoma (37). Consistent with these results, MFGE8 have been found to play a critical role in breast cancer pathobiology and clinical prognosis (38, 39). Furthermore, MFGE8 knockdown significantly inhibited both migration and proliferation of tumor cells, attenuating their tumorigenic properties (40). As for HAPLN3, it has been identified as a novel diagnostic and prognostic biomarker for prostate cancer (41). Besides, the expression of HAPLN3 was shown to be significantly higher in breast cancer tissues compared to the normal breast tissues. It was associated with the metabolism dysregulation, mobility, and migration of cancer cells in TNBC (42, 43). These imply the value of *MFGE8-HAPLN3* in guiding diagnosis and treatment choices in cancer.

Inevitably, there are some limitations in our study. First, we could not distinguish fusions occurring at the transcriptome level from those at the genome level by algorithms we used. Future studies are required for a comprehensive understanding of the selected fusions. Second, due to the small sample size, it is difficult to make any rigorous conclusions regarding the subtype-specific distribution of fusion transcripts. Studies are needed to point out the frequency of *MFGE8-HAPLN3* in different subtypes to distinguish patients with clinical benefit from targeted therapy in the future. Furthermore, the predicted ORF of *MFGE8-HAPLN3* and its pathologic and therapeutic role in TNBC requires further experimental validation.

In conclusion, our large-scale analysis revealed a number of fusion transcripts in TNBC for the first time. Remarkably, *MFGE8-HAPLN3* could be a candidate biomarker and potential therapeutic target in TNBC. Further investigations are required to elucidate the underlying mechanisms and their biological functions.

## Data Availability Statement

The data sets presented in this study can be found in online repositories. The names of the repository/repositories and accession number(s) can be found in the article/**Supplementary Material**.

## Ethics Statement

Our study was approved by the independent ethics committee/institutional review board of Chongqing University Three Gorges Hospital. Written informed consent for participation was not required for this study in accordance with the national legislation and the institutional requirements.

## Author Contributions

M-YW: conception of the work, data collection, data analysis and interpretation, drafting the article, critical revision of the article, and final approval of the version to be published. MH and C-YW: conception of the work, data collection, critical revision of the article, and final approval of the version to be published. X-YT, J-GW, Y-DY, XX, and C-WG: data collection, critical revision of the article, and final approval of the version to be published. All authors contributed to the article and approved the submitted version.

## Funding

This work was supported by the Natural Science Foundation Project of Chongqing, China (cstc2016jcyjA0338).

## Conflict of Interest

The authors declare that the research was conducted in the absence of any commercial or financial relationships that could be construed as a potential conflict of interest.
