# Supplementary material for: Transcriptome Analysis Reveals MFGE8-HAPLN3 Fusion as a Novel Biomarker in Triple-Negative Breast Cancer
Source: Front Oncol. 2021 Jun 15;11:682021. doi: 10.3389/fonc.2021.682021 (PMC8239224; doi:10.3389/fonc.2021.682021)
Supplement: Supplementary file 1 [file Table_1.docx]

**Supplementary Table 1. Fusion transcripts detected in TNBC.**

| AAK1_NFU1 | ACADL_KANSL1L | ACTL8_PADI4 | ADCY3_PTRHD1 | ADNP2_ATP9B |
| --- | --- | --- | --- | --- |
| AGO2_TRAPPC9 | **ANKLE2_POLE** | **ANO1_XRRA1** | **AP5B1_AHNAK** | **AP5S1_MAVS** |
| ARHGEF17_SHANK2 | **ARRDC1_EHMT1** | **ATP2C1_NEK11** | **BAZ2B_WDSUB1** | **BPTF_CLTC** |
| C15orf57_CBX3 | **C1RL-AS1_CLSTN3** | **C21orf58_MACF1** | **CA8_YPEL2** | **CBX1_PLEKHA2** |
| CCDC88C_FRMD8 | **CDC42BPA_PIK3C2B** | **CDH1_SNTB2** | **CEP350_AXDND1** | **CEP350_TOR1AIP1** |
| CHL1_TMEM123 | **CIRBP_C19orf24** | **CLN6_CALML4** | **CLN8_ARHGEF10** | **CLTC_VMP1** |
| CNOT11_ANKRD36 | **COL7A1_UCN2** | **COLGALT1_ZBTB45** | **COMMD1_B3GNT2** | **COPS3_DDX11L1** |
| COPS3_DDX11L5 | **COPS3_DDX11L9** | **CREBBP_TMEM200C** | **CREBBP_TRAP1** | **CSRP1_LAD1** |
| CTBS_GNG5 | **CTSC_RAB38** | **CTTNBP2_AKAP9** | **CXADR_BTG3** | **DENND4C_PTPRD** |
| DHTKD1_SEC61A2 | **DSE_FAM26F** | **DSTN_RRBP1** | **DSTYK_KLHL4** | **EEF1A1_RMRP** |
| EEF1D_NAPRT1 | **EIF3H_TRPS1** | **EIF4G3_HP1BP3** | **ELF5_PAMR1** | **FAM115A_PRSS37** |
| FAM214A_MYO5A | **FARSB_VSNL1** | **FAT1_MTNR1A** | **FOXJ3_RIMKLA** | **FREM1_CER1** |
| GKAP1_KIF27 | **GNAQ_RPGRIP1L** | **GPBP1L1_C1orf87** | **GSS_UBR2** | **HAO2_HSD3B2** |
| HARS2_ZMAT2 | **HDAC3_DIAPH1** | **HORMAD1_GOLPH3L** | **HUWE1_PHF8** | **IL1R1_PDPR** |
| IRF6_C1orf74 | **ITGB5_SLC12A8** | **ITGB6_RBMS1** | **KCNN4_LYPD5** | **KDM4B_MAST1** |
| KIAA0232_TBC1D14 | **KIAA0319L_CLSPN** | **KIAA1109_CAMK2D** | **KLHL24_NPSR1-AS1** | **KMT2A_TMEM25** |
| LACTB2_TRPM1 | **LEPREL2_CD9** | **LIN54_COQ2** | **LPP_TPRG1** | **LRP6_SENP6** |
| MED22_SURF6 | **METAP1_IDO1** | **MFN1_GFM1** | **MFSD7_ATP5I** | **MLLT4_LYRM2** |
| MMP2_EXOC3L4 | **MSMB_NCOA4** | **MTDH_C15orf26** | **NADSYN1_MRPL48** | **NELFB_SUMF1** |
| NFIC_CELF5 | **NFX1_SPINK4** | **NMRAL1_ANKS3** | **NSUN4_FAAH** | **NXN_GLOD4** |
| PLA2R1_RBMS1 | **PPP1R13L_ERCC1** | **PPP1R2_DLG1-AS1** | **PPP3CA_RYBP** | **PRMT7_NUP155** |
| PROM1_TAPT1 | **PSPC1_PAN3** | **PTGIS_B4GALT5** | **PVT1_SNTG1** | **RAB1A_DGUOK** |
| RAB1A_ZNF638 | **RAB6A_C2CD3** | **RANBP10_ENKD1** | **RGL1_EXOC4** | **RNASEH1_ADI1** |
| RNASET2_RPS6KA2 | **RNF139_NDUFB9** | **RNU6ATAC_WDR74** | **RPPH1_RMRP** | **RRM2_C2orf48** |
| RSBN1_NFIA | **SAV1_MAP4K5** | **SESTD1_ITGA4** | **SMURF2_CEP112** | **SNX9_TULP4** |
| SOBP_AIM1 | **SPTLC2_ISM2** | **SRPK2_PUS7** | **ST7_ARF5** | **STYXL1_TMEM120A** |
| SUZ12_ANKRD36 | **SYT8_TNNI2** | **TANC1_PKP4** | **TBC1D9_LSM6** | **TC2N_FBLN5** |
| TEX2_KCNMB3 | **TMEM165_KIT** | **TMEM19_MUC19** | **TMEM241_ANKRD29** | **TNFSF10_NCEH1** |
| TNPO2_STX10 | **TOMM40_IGSF23** | **TOPORS_DDX58** | **TP63_TPRG1** | **TRADD_B3GNT9** |
| TRIM2_FHDC1 | **TRPS1_EIF3H** | **TSG101_GRM5** | **UBE2J2_MRPL20** | **UBXN2A_CEP85** |
| URI1_VSTM2B | **USP10_CDH13** | **VGLL4_TAMM41** | **VPS13D_DRAXIN** | **WNK1_ERC1** |
| WTAP_ACAT2 | **WWTR1_DAPK2** | **YARS_NDC1** | **ZBTB37_RABGAP1L** | **ZCCHC11_PPP1R8** |
| ZMYM2_DDX11L1 | **ZMYM2_DDX11L10** | **ZMYM2_DDX11L5** | **ZMYM2_DDX11L9** | **ZNF180_CEACAM20** |
| ZNF606_C19orf18 | ACSM1_GINS2 | AZGP1_GJC3 | B4GALT1_DENND4C | BHLHB9_LINC00630 |
| EEF1DP3_FRY | FIGLA_ADD2 | GLYCTK_DNAH1 | LDLRAD3_PRR5L | MBD1_CCDC11 |
| MFGE8_HAPLN3 | MOB3B_EQTN | OTUD6B_LRRC69 | PMM2_ABAT | PPCS_CCDC30 |
| PRKAA1_TTC33 | RAB9A_OFD1 | RNF213_SLC26A11 | SLC12A7_TERT | SLC25A16_DNA2 |
| SLC35A3_HIAT1 | SLC4A7_NEK10 | VAX2_ATP6V1B1 | ZNF765_TPM3P9 |  |

Bold and underline indicate tumor-specific fusion transcripts (N=166).

**Supplementary Table 2. Comparison of frequency of fusion events between tumor tissue and para-tumor tissue.**

|  | Tumor tissue | Para-tumor tissue |
| --- | --- | --- |
| All fusions | 189 | 37 |
| Specific fusions | 166 | 14 |
| Min fusions per sample | 1 | 1 |
| Max fusions per sample | 20 | 5 |
| Mean fusions per sample | **4.106^*^** | 1.807 |

* p < 0.05

**Supplementary Table 3. Frequency of fusion transcripts in different molecular subtypes.**

| All (N = 66) |  | BLIS | IM | MES | LAR |
| --- | --- | --- | --- | --- | --- |
|  | N | 28 | 15 | 8 | 15 |
| All Fusions | FTs per Sample | 1~14 | 1~20 | 1~5 | 1~11 |
|  | Average | **5.04*** | 3.93 | **2.38*** | 3.47 |
| Redundant Fusions | FTs per Sample | 0~5 | 0~4 | 0~2 | 0~4 |
|  | Average | **2.11*** | 1.27 | **0.88*** | 1.27 |
| Private Fusions | FTs per Sample | 0~10 | 0~16 | 0~5 | 0~7 |
|  | Average | 2.93 | 2.67 | **1.5*** | 2.2 |

FTs, fusion transcripts; BLIS, basal-like immune-suppressed; IM, immunomodulatory; MES, mesenchymal-like. LAR, luminal androgen receptor;

* *p* < 0.05
